# Medical Causation and Expert Testimony: Allergists at this Intersection of Medicine and Law

**DOI:** 10.1007/s11882-012-0294-z

**Published:** 2012-08-29

**Authors:** Howard M. Weiner, Ronald E. Gots, Robert P. Hein

**Affiliations:** 1 Medical Assessment Institute, Inc, 2385 NW Executive Center Drive, Boca Raton, FL 33431-8510 USA; 2 International Center for Toxicology and Medicine, Rockville, MD 20850 USA; 3 Fowler, Hein, Cheatwood & Williams PC, 2970 Clairmont Road NE Suite 220, Atlanta, GA 30329‐4414 USA

**Keywords:** Causation, Causation analysis, Medical/legal, Source identification, Differential diagnosis, Differential diagnosis vs. Causation

## Abstract

Clinical practice always necessitates proper diagnosis and correct treatment. For most clinical fields, determining the cause of the illness is irrelevant to the intervention. An oncologist, for example, has no need to explore the “cause” of the patient’s lymphoma. Allergists, by contrast, have tools and the need to examine the relevant allergen which is the putative “cause” of the patient’s allergic symptomatology. In the context of a legal claim, the “cause” of the symptoms or disorder is central, because it determines financial responsibility. However, in the case of an allergic disorder and identified allergen, a claim requires more. Whose allergen? Where did it come from? These are crucial questions that must be answered. This paper explores the approaches to causal assessment which are important for the clinical allergist as he/she navigates the interface between clinical practice and legal proceedings. Its purpose is to help the allergist understand that interface, and to be prepared to enter an unfamiliar legal arena.

## Introduction

Diagnosis, treatment, and prevention are the primary roles of the practicing physician. What is wrong with the patient? How do I treat him? How do I keep him well? Causal analyses are secondary, and, for most clinical fields, entirely unimportant. It does not much matter to an oncologist, for example, what caused the patient’s leukemia. The patient will frequently ask or supply his own theories, but the therapeutic intervention is determined by the diagnosis, not the etiology.

By contrast, legal disputes, as opposed to clinical ones, almost always involve causal questions and demand causal answers. Did the contaminated drinking water cause the cancer? Did formaldehyde from the insulation cause the chronic rhinitis? Did the mold in the home arising from a leaking roof or window cause the child’s asthma? Did the asbestos in the workplace cause the lung cancer or was it the 40 pack-years of cigarette smoking? The answers to such questions are central to asserting liability claims against the polluter, the insulation installer, the home builder, or the asbestos producer in a court of law.

Allergists, as distinguished from oncologists, rheumatologists, and orthopedic surgeons frequently address the question, “What caused the patient’s allergy?” This clinically important causal question, when addressed, may require fastidious history taking and diagnostic approaches such as allergy skin testing and other diagnostic inhalational/dermal/oral/subcutaneous challenges. Since not all patients who are skin tested demonstrate clinical concordance with the history elicited, that question may have a presumptive, but unproven answer. For example, was it allergic versus vasomotor rhinitis? Or was it allergic versus irritant-induced asthma. In the context of a lawsuit, an allergist’s working diagnosis is insufficient. A supportable, final diagnosis is necessary.

Another, less familiar, causal question is also demanded in legal proceedings. In addition to a determination of which agent or agents are responsible for symptoms, a liability claim usually requires expert opinions about the source of that agent. Where did it come from? Who was responsible? Historically, patients rarely ask: “whose pollen, which latex product, or where did the mold come from?” That changed for allergists when various environmentally-encountered agents acquired the dual role of both potential allergens and the subject of a lawsuit. This placed a new demand on allergists who were now asked to assist a patient by offering expert medical opinions about the strength of association or link between the suspected source of the allergen and the medical symptoms or illness observed.

Moreover, the allergist must be prepared to explain the basis for that attribution in part relying on other experts who may have identified how, when, and why the suspected environmental conditions or agents occurred. But the allergist must be able to comfortably link those findings to the clinical disorder. He may be asked to support that causal nexus with these types of questions: “Doctor, given the airborne level of mold spores found and the description of the water damage, was the duration and extent of potential exposure sufficient to have caused the patient’s asthma?” “What is your basis for that conclusion?”

The purpose of this discussion is to elucidate, for allergists, this interface between clinical responsibilities of the doctor and legal demands of the patient as claimant and the court system. Even for clinical allergists who have no intention of or desire to be formally hired as experts, causal questions involving both the documentation of the specific allergy and the assessment of the source of the allergen(s) are important to the patient who is also a claimant. For an allergist hired by the plaintiff or the defendant’s attorney as an expert witness for the purpose of establishing causation, both questions, their answers, and a critical analysis of the bases for those answers are the linchpins of the expert’s role.

In the discussion to follow, we shall attempt to delineate those questions which must be asked and answered by the allergist as expert witness. We will begin with the medical causation issues, followed by the source identification issues. We will then present the legal requirements and show how and why physicians can be led astray or have their testimony excluded by judges for lack of a proper methodology or foundation. The goal of this article is to provide the allergist with the necessary information to assure reliability and admissibility of his testimony so it is helpful to the court and to minimize the risk of a disconcerting experience.

## Medical Causation Analysis

The science of causation assessment is well accepted and generally recognized. This is the field which refers to the scientific methods by which, for example, a microbiological agent, chemical, allergen, irritant, drug, or medical device can be causally connected to or ruled out as a cause of disease.

To determine whether a causal relationship exists between a given agent or substance and adverse health effects, it is essential that proper causation methodology be applied. Methods of assessing causal relationships are not matters of opinion. Rather, methods for investigating causal relationships between agents and illnesses have been well delineated in the scientific literature [1–14].

One simplified overview of the components of a proper causal analysis was published by one of the authors who called the methodology the “Does, Can, Did” approach [6]. “Does” refers to the diagnosis: from what ailment(s) does the patient suffer? “Can” refers to the capability of the alleged causal agent to actually cause the identified disorder(s). And “Did” refers to the result of specific investigation carried out to link the putative causal agent(s) to the specific individual patient. The first component, “does,” is the diagnostic element. This, of course, is familiar to the clinician because it is the bedrock of clinical as well as medical legal practice. However, the very fact of a claim against an employer or a presumed tortfeasor (a person who has committed a wrongful act) adds a confounder to the diagnostic encounter which can lead the practitioner astray if not considered. The next two elements: “Can” and “Did” have, since the introduction of these descriptive terms, been named by the courts as “general” and “specific” causation. Numerous decisions concerning expert testimony and the very admissibility of an expert’s opinion have turned on these causal elements.

## General Causation

In order to arrive at cause and effect conclusions regarding exposures and health effects or disease processes, certain elements are crucial. First, there must be scientific knowledge that the agent is capable of causing the health effects or conditions in question. This element has been called “general causation.” Second, there must be sufficient information available to conclude that the agent was causal in this specific individual. This is called “specific causation.”

In many specialty areas, general causation is established through epidemiologic studies. The method by which those studies are combined and weighed to assess causal probabilities has been most closely connected to a speech by Sir Bradford Hill in 1965 [10]. Dr. Hill was speaking about the growing numbers of studies which linked lung cancers to cigarette smoking and asked: how many studies of what quality are necessary before we can assert a causal relationship? The principles which he laid out in that speech are commonly called (though not exactly correctly) “Hill’s Criteria” and they lay the foundation for much of general causation oversight in the courts today.

There is much more medical knowledge about the cause of common allergic diseases than about causes of cancer. Consequently, when epidemiologic studies have not established chemical X to be a carcinogen, it is methodologically improper for an oncology expert to assert that it caused his patient’s cancer. For pure allergy matters, by contrast, clinicians properly rely on medical evidence which is not always supported by epidemiological studies. The chemical nature of a substance, its potential as an allergen, and clinical experience in many patients is often sufficient. However, allergists also deal with non-allergic responses which present general causation conundrums requiring epidemiology and basic toxicology in order for a general causal assertion to be justified.

Consider, for example, a claim of irritation associated or suspected with a workplace chemical, new furniture or carpet, fresh paint, or a perfume. Consider the progression of this irritation into Multiple Chemical Sensitivity Syndrome, a medically unproven condition. Before the allergist asserts that the patient’s symptoms and/or clinical manifestations were caused by the irritant effects of the chemical alleged, he/she had better know that that chemical is, in fact, an irritant and at what levels such effects occur. This can generally be found in the basic toxicological literature [15–17]. So, while formaldehyde can be mildly irritating at between 0.1 and 0.3 parts per million, benzene, toluene, and most other common organic chemical constituents of paints, gasoline, and perfume are not [18, 19]. Alcohols, simple aliphatic hydrocarbons, and a simple benzene ring generally have mild-to-no irritating properties, whereas aldehydes and ketones are significantly more irritating. The allergist must assume that, if he asserts the existence of a causal link between symptoms and the alleged chemical irritant in the context of his patient’s claim, he will be questioned about basic toxicological principles pertaining to irritation and chemical structure. It is, after all, fair game for the opposing attorney to ask about and to challenge the methodology which led the physician to reach his general causal conclusion. And, in this case, that methodology is premised upon the basic toxicological/irritant properties of the relevant chemicals.

Certain general causation questions of dubious established connection may confront the allergist. An example may be the multi-organ, multi-system complaints which the patient has attributed to mold or mold toxins. Everything from cognitive dysfunction to gastrointestinal disturbances fall into the category of common symptoms with little general causal scientific support [20–26]. The allergist who affirms the patient’s causal belief in such matters can expect probing questioning about the scientific merits of that claim by a well-prepared attorney.

## Specific Causation

Once the general causation question is asked and answered affirmatively, the physician can move on to the specific causation issue. Now that we have established the plausibility of the contended causal relationship, we must answer the question: “Has it actually occurred in this patient?”

Specific causation is made up of several essential subparts: (1) the dose must have been sufficient to have caused the claimed adverse health outcome; (2) the temporal relationship must have been correct; (3) if the claimed disorder is allergenically-based, the individual must have demonstrated allergic sensitization to the agent(s) at issue; and (4) other causes must have been considered and ruled out.

**Fig. 1:**
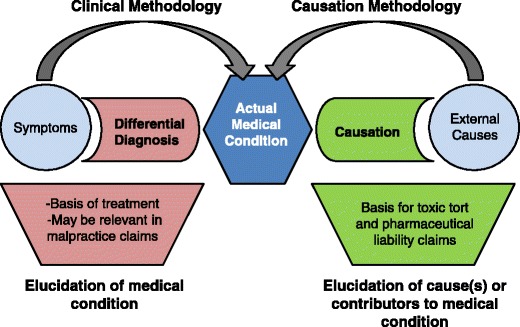
Differential diagnosis versus causation analysis

Figure 1 illustrates the distinction between the diagnostic methodology and the causation assessment methodology. An evaluation begins with a quest for the diagnosis utilizing a standard differential diagnostic approach. Causation assessment, a distinct and separate process, follows utilizing the principles previously delineated.

## The Role of Differential Diagnosis in Causal Analysis

From a scientific/medical perspective, the law requires that the expert who is providing a medical opinion on causation follow a methodology consistent with the methodology utilized by his peer group. In the past few years, a growing number of courts have, quite improperly, in our opinion, taken that requirement to mean that the process of differential diagnosis would properly lead to both a diagnosis and a causal conclusion. Figure 1 illustrates why that is not the case. If it were true that the diagnostic process uncovered an external cause with no further analysis required, a physician could ascribe any cause of his/her choosing to an ailment, whether or not there was even a modicum of scientific support for such a connection. He could simply assert that the patient suffers from a disorder of his own invention and that his conclusion was the result of having excluded all other conditions in the differential diagnosis. A causal conclusion requires an analysis quite separate from a diagnosis. The fact that an oncologist establishes a diagnosis of acute myelogenous leukemia and states that it was due to benzene exposure does not mean that he/she has supported that causal attribution. Simply asserting that the patient has AML caused by benzene does not make that assertion correct. To be correct, the general and specific methodological approaches necessary for specific causation delineated above must have been followed.

The same can be said for a diagnosis by an allergist. One might diagnose probable occupational asthma rather than exacerbation of non-occupational adult-onset asthma, but such a conclusion needs more that a history alone to establish and testify to the probability of that causal attribution. Recognizing this dichotomy between diagnosis and causal assessment, some courts have defined the processes more like the one illustrated in Fig. 1 and called these separate analyses “differential diagnosis” and “differential etiology,” another name for causation assessment.

While it is indisputably true that the result of the differential diagnostic process quite properly leads physicians to a clinical diagnosis, it is certainly not the case, except in rare instances, that it leads to causal conclusion. Even for allergists, there are two processes. First, the allergist must determine whether the red eyes are the result of irritation, infection, or allergy. Once allergy becomes the diagnosis, only then can the etiology (which allergen?) be sought and established.

## Analyzing the Exposure Source

Identifying the source of the exposure is probably the most vexing issue of all the causal questions which arise in a lawsuit. Allergists are more attuned than most specialists to this concept. They often need to provide allergy-mitigation recommendations which can lead to generic protection by, say, bed covers to reduce the load of dust mites.

However, in the context of a claim in which a roof leak is linked by the patient or his/her lawyer to respiratory allergies, the allergist must demonstrate an intense investigation of the source of the allergen in order to establish a legally acceptable causal conclusion. Often the allergist will accept the patient’s attribution, converting and transforming the patient’s perceived cause to a medically espoused and ratified one. The patient said: “My symptoms started with the roof leak and mold grew in the house; therefore, mold in the house from the roof leak caused my illness.”

However, before the allergist can testify to such a causal attribution, he/she must anticipate and consider how the questioning by the opposing attorney will proceed: “How long did the roof leak and how much water damage was there?” “How much mold grew and where was it?” “What genera and species of mold were found in the air?” “How large an area did the mold cover?” “How representative of the entire living space was the swab sample you reviewed?” “If you are claiming that this was a ‘wet/damp’ space, how wet was it and how much space was wet and damp?” and “Did you review the indoor air evaluation reports or perform a site visit?” These are only a few of the reasonable questions that will likely be asked of a physician or any other expert who links a source, in this case a roof leak, that allegedly led to mold growth that allegedly led to a clinical outcome.

## Patient/Claimant and Physician Confounders

It is not unusual for patients visiting the allergist to come with some idea of the “cause” of their problems, whether it is a specific food, seasonal outdoor allergens, a damp home, workplace, perfume, or other environmental irritants. It is a near certainty that, if a legal claim has prompted the visit to the allergist’s office, the patient will think he or she “knows” the cause of his/her complaints. It is also a given that a refutation of a patient’s causal attribution, if not confirmed by clinical evaluation, is more readily accepted by the patient who is not a claimant than by one who is. The latter, after all, is driven by two impulses—to get medical help and to succeed in a pursuit of the claim.

The patient’s suspicions about the cause of his or her illness, combined with the desire to hold someone liable for causing that problem, inevitably leads to certain reporting biases, whether intentional or subconscious, which commonly color the clinical history. Another bias in claims-related matters is a form of attribution bias which comes from fear or worry about environmental hazards. For example, the explosive proliferation of print and broadcast media, as well as internet attention, to “toxic mold” has contributed to the public’s attention to and fear of even minute and harmless amounts of indoor contaminants.

Physicians, too, have, at times, contributed to patients’ beliefs that they are ill and that certain environmental factors are the cause. By misattributing symptoms to a cause or diagnosis of questionable validity, a physician can encourage and reinforce a patient’s notions and exacerbate the illness through an iatrogenic effect [27]. All these issues, reporting bias, hazard perception, and iatrogenic contribution, have been studied and documented [28–36]. Patient and physician biases contribute both to the diagnostic interaction between the clinician and the patient/claimant, as well as to the causal attribution and analysis by the clinician.

## Legal Causation Analysis

The determination of legal causation of a medical illness requires expert medical testimony on the medical and environmental cause of injuries. This commonly includes a detailed toxicological, epidemiological, and/or, at times, an industrial hygiene-supported causal assessment. Whether or not the medical opinion is admissible depends on whether it meets court-accepted standards of reliability. In many states, and in federal courts, that standard is known as the “*Daubert* standard.”

## Admissibility of Expert Testimony

The specific legal rule in federal court pertaining to whether an expert’s testimony is admissible is found in Rule 702 of the Federal Rules of Evidence. The rule also applies to other experts as well as to physicians.

A witness who is qualified as an expert by knowledge, skill, experience, training, or education may testify in the form of an opinion or otherwise if:

1. The expert’s scientific, technical, or other specialized knowledge will help the trier of fact (a judge or jury) to understand the evidence or to determine a fact in issue;
2. The testimony is based on sufficient facts or data;
3. The testimony is the product of reliable principles and methods; and
4. The expert has reliably applied the principles and methods to the facts of the case.

## *Daubert* Criteria

In 1993, the U.S. Supreme Court clarified Rule 702 and the admissibility of expert medical testimony in a case known as *Daubert v. Merrell Dow Pharmaceuticals, Inc*., 509 U.S. 579 (1993).The *Daubert* case set out four factors used by the courts in determining whether an expert witnesses’ theory, technique, or opinion on the causing of a medical illness is scientifically or medically valid such as to allow it to be considered in the case. This standard is now used by courts to determine whether a physician’s opinion on medical causation is admissible in evidence for consideration by the court or the jury. The *Daubert* factors and Rule 702 provide the standard for determining legal causation in a civil lawsuit.

Those four primary, but non-exclusive, *Daubert* factors are:

1. Can or has the medical or scientific theory, knowledge, opinion, or technique been tested?
2. Has the medical or scientific theory, knowledge, opinion, or technique been subjected to publication and peer review?
3. Is there a known or potential rate of error for the method, knowledge, opinion, or technique?
4. Is the medical or scientific theory, knowledge, opinion, or technique accepted by the majority of other medical or scientific peers within the relevant medical or scientific community?

These four *Daubert* factors are not the only ones a court may consider, and the fact that the doctor’s opinion does not meet one of the criteria does not necessarily result in exclusion of the testimony. The lack of peer review or publication will not preclude admissibility if the doctor’s opinion is supported by widely-accepted scientific or medical knowledge. The fact alone that a physician has extensive medical training and several board certifications in specialty areas will not make his or her opinion legally admissible or reliable if the rest of the doctor’s peers do not accept that opinion as mainstream. The physician’s specialties may make him or her qualified as an expert, but do not make the medical opinion on causation admissible.

In the *Daubert* case, the plaintiff’s attorney sought to prove that Bendectin taken during pregnancy caused birth defects. There were eight experts prepared to testify that Bendectin taken during pregnancy both can (general causation) and did (specific causation) cause the child’s birth defects. The Plaintiff sought to prove the causation using in vitro, animal, and epidemiological studies. The U.S. Supreme Court did not rule on the admissibility of the physician’s opinions, but did establish the guidelines for federal courts in determining the relevancy and reliability of such medical opinions and scientific evidence.

Frequently, the state of medical/scientific knowledge and research has not advanced far enough to warrant widespread acceptance. The fact that the doctor feels passionately about the medical evidence is not sufficient for the court to accept the opinion on medical causation if the rest of the medical community itself is not sure. While future research and studies may well validate the physician’s opinion on causation, sometimes the state of medical knowledge has not yet progressed to the comfort level of the legal system. Yet, the legal system mandates a scientific disposition at this point in time. This demand may be unsettling for the physician acting as an expert witness.

## An Attorney’s Perspective: Confusion of the Courts in Their Use of “Differential Diagnosis”

From an attorney’s perspective, the clinical treatment process of rendering a diagnosis to identify the illness causing the patient’s symptoms (a differential diagnosis) is often confused by both doctors and attorneys with the process of determining the specific scientific or medical cause of the illness diagnosed (causal assessment). Many court opinions have mixed the two methodologies by sometimes equating a causal assessment to a differential diagnosis. In other words, the courts have conflated the two methodologies when discussing them in written court opinions. In reality, the courts are looking for, and want a causal assessment with, a high degree of empirical or scientific and medical support and proof, not a differential diagnosis.

The goal of the physician in performing a differential diagnosis is to determine what illness is causing the symptoms in order to treat the patient. Determination of the underlying cause or what actually produced the condition is generally not critical in most clinical interactions. However, a claim requires a causal attribution. The court must have a thorough explanation of whether the doctor’s opinion on causal assessment is “ground[ed] in the methods and procedures of science,” by considering the physician’s testimony using a “two-pronged test of relevancy and reliability in the context of scientific evidence.” [37••]. Relevancy refers to testimony that is likely to be helpful to the court and prove a key issue in the case. Reliability refers to testimony which meets the *Daubert* criteria.

When determining *during the trial* whether a defendant is liable for causing an injury (or illness), the judge or jury must find by a “preponderance of the evidence” that the defendant caused the harm, exposure, or injury to the plaintiff. The causal attribution must be established to a degree or medical probability or certainty, clearly more than a coin toss, and more than 50 %.

But *Daubert* motions are heard *before* trial and must offer the court an even higher degree of proof of causation before the physician’s opinion is admissible. Each element used by the expert must be established and generally accepted. For the scientifically-based underpinnings (as needed for “can” or general causation), the degree of certitude must follow those generally required in science, or, by custom, greater than 95 % (*p* value of < .05). Once that foundation (e.g., that x is an established cause of y) is known with this degree of probability, the doctor then may testify to opinions that all of the causation elements were met—e.g., exposure was sufficient, temporal relationships were correct, and other causes were ruled out. Those elements of testimony, needed for specific causation, require, not 95 % certainty, but a preponderance of the evidence standard of greater than 50 %. Even attorneys have difficulty getting a clear understanding of this distinction between legal and scientific causal probabilities. In fact, in the interest of “catching” the witness, they may ask whether his testimony is stated to scientific or legal certainty, implying that the former is more stringent than necessary. The answer to that question is actually, “Both.” Scientific certainty is needed to solidify general causation—that such a relationship is known scientifically to be true. Legal certainty (more probable than not) follows for the remainder of the causal questions.

So, in essence, a *Daubert* inquiry is looking for causal assessment of a high order from the physician who testifies as to what environmental condition, exposure, biological process, or other explanation led to the injury or harm that forms the basis of the lawsuit. In the context of a *Daubert* inquiry, it is safe to say that “legal causation” depends heavily on a reliable and scientifically established medical “causal assessment.”

Ultimately, the judge acts as a “gatekeeper” in deciding to let the doctor’s opinion in or to keep it out of consideration. The legal vehicle most often used for addressing admissibility of the physician’s opinion on medical causation is a *Daubert* Motion to Exclude Evidence.

## An Attorney’s Perspective: Using a Hypothetical Medical and Legal Case

In the brief example which follows, we shall illustrate the types of questions which an expert may face by opposing counsel. These questions will vary depending upon the expert’s specific opinions in the case. They might, for example, challenge the diagnosis, the existence of an allergy, the alleged allergenic agent, or the source of that agent. Questions will also be posed which challenge the expert’s qualifications and motivations.

Consider a 60 year-old male who rented an apartment until his new condominium was completed. He has a 40 pack-years cigarette smoking history, but never had respiratory symptoms until having lived in this apartment for 2 months. He noticed a significant water leak in the guest bedroom closet and visible mold growth limited to its ceiling. He went to his internist for treatment of his shortness of breath and cough, wondering if he had developed asthma from mold in the apartment.

Assuming that he became a claimant in a lawsuit against the apartment owner, serving as the patient/claimant’s expert witness, an allergist could be asked these sample questions. They appear in a seemingly random order, a manner commonly employed by an experienced cross-examiner.

1. What respected, authoritative, and widely-accepted medical studies and research support the link between mold and this individual’s medical conditions?
2. Is the link a strong or weak association?
3. What is the typical period of exposure necessary to produce the disease?
4. Has the claimant shown any IgE reaction to the suspected allergens as confirmed by radioimmunoassay, skin, or other tests?
5. Did the physician (or an environmental expert performing accepted testing methodologies) sample the air in the suspected bedroom closet and perform such studies throughout the apartment to establish the specific mold species and exposure level?
6. What was, in fact, the dose level of specific molds to which the claimant was exposed?
7. Did the results of the allergy tests performed confirm allergic sensitization to the same specific molds found through environmental testing in the bedroom closet and apartment?
8. Did the allergist review the previous medical history of the claimant prior to his renting the apartment?
9. Can the allergist compare the claimant’s exposure to mold or other allergens in the outdoor environment versus exposure in the indoor environment?
10. What levels of each of those molds are commonly found outdoors?
11. Were there any baseline air samples taken of the bedroom closet, apartment, and outside air in the claimant’s apartment?
12. Is the allergist sufficiently comfortable with the validity and methodology employed by the environmental consultant who conducted sampling to rely on those test results for providing a definitive statement of the specific allergen, exposure, dose, and likely response?
13. Is there a known rate of allergic response associated with the exposure and dose alleged to have occurred?
14. How much of the allergist’s opinion is based on claimant-reported exposure conditions and subjective symptoms versus the physician’s personal observation and knowledge of the underlying environmental conditions, pre-existing medical history, and objective findings?
15. Has the allergist personally designed and performed any medical studies using the scientific method (control vs. non-control groups) to confirm the validity of the opinion that the indoor apartment exposure to the specific molds in question under the extrapolated exposure and dose data are sufficient to a high degree of reliability in causing the specific medical illness and symptoms observed, after accounting for all confounding variables.
16. Has the allergist published the data or performed any studies and reviews of medical literature to confirm the proposed environmental or biological cause of the illness?
17. What position do the majority of clinical allergists take with regard to the medical literature, studies, and opinion of the testifying allergist on the biological and medical cause of this illness?

## An Attorney’s Perspective: The Clinician in the Legal Arena

Clinical practice seeks scientific and medical accuracy without which the best treatment cannot be provided. A civil claim, by contrast, provides a forum for the resolution of disputes and most legal scholars argue (to the surprise of inexperienced medical experts) that it is not a truth-seeking forum at its core. The opposing attorneys are advocates. Their interest is in winning, not in enlightening the court. This distinction is felt by the expert when he/she undergoes intense examination, whether in deposition or trial. The intent of those questions, all designed to erode the expert’s credibility, may be to prepare to file a *Daubert* motion to have the testimony excluded or simply to win over a judge or a jury.

How can the allergist, as a witness, prepare for such an encounter? The first rule is to be honest, accurate, and consistent in his/her opinions. Attorneys will attempt to impeach the expert’s integrity by demonstrating inconsistencies in his/her prior opinions referenced in scientific articles, depositions, and court testimonies. The expert must not stray outside his/her areas of expertise. He/she must be sure that the facts stated are both sufficient and reliable in supporting the proffered position. The second is to know those facts and their supporting bases well before encountering opposing counsel. Finally, while it may be difficult, the witness should remain calm and rational in the face of what may seem to be foolish and/or insulting questions. The expert is not a combatant but rather a privileged participant [38•].

## Conclusion

This article has highlighted essential points, nuances, and considerations for the practicing physician who will be asked to render an expert medical opinion on the cause of a patient’s medical illness or condition. As a practising allergist/clinical immunologist (H.M.W.), physician/toxicologist (R.E.G.), and an experienced civil litigation attorney (R.P.H.), the authors have provided insights into the challenges of rendering a diagnosis, determining medical causal assessment, and offering a legal causation analysis. The primary focus was to help elucidate the unfamiliar idiosyncrasies of a legal proceeding. As a patient advocate or medical expert, participation in the legal system is an opportunity to place state-of-the-art science into case law.
